# Zinc: a potential star for regulating peritoneal fibrosis

**DOI:** 10.3389/fphar.2024.1436864

**Published:** 2024-09-04

**Authors:** Jian Li, Xinyang Li, Yangwei Wang, Lingfei Meng, Wenpeng Cui

**Affiliations:** Department of Nephrology, The Second Hospital of Jilin University, Changchun, China

**Keywords:** peritoneal dialysis, end-stage renal disease, zinc, oxidative stress, peritoneal fibrosis

## Abstract

Peritoneal dialysis (PD) is a commonly used renal replacement therapy for patients with end-stage renal disease (ESRD). During PD, the peritoneum (PM), a semi-permeable membrane, is exposed to nonbiocompatible PD solutions. Peritonitis can occur, leading to structural and functional PM disorders, resulting in peritoneal fibrosis and ultrafiltration failure, which are important reasons for patients with ESRD to discontinue PD. Increasing evidence suggests that oxidative stress (OS) plays a key role in the pathogenesis of peritoneal fibrosis. Furthermore, zinc deficiency is often present to a certain extent in patients undergoing PD. As an essential trace element, zinc is also an antioxidant, potentially playing an anti-OS role and slowing down peritoneal fibrosis progression. This study summarises and analyses recent research conducted by domestic and foreign scholars on the possible mechanisms through which zinc prevents peritoneal fibrosis.

## 1 Introduction

As the global burden of end-stage renal disease (ESRD) grows, the demand for cost-effective renal replacement therapies rises. Common renal replacement therapies include haemodialysis (HD), peritoneal dialysis (PD) and kidney transplantation. PD is widely used in patients with ESRD worldwide, especially in developing countries, due to its simplicity, safety, effectiveness and suitability for home treatment. Currently, over 272,000 patients worldwide undergo PD, representing approximately 11% of the global dialysis population (Himmelfarb et al., 2020). Asia currently has the highest number of patients undergoing continuous ambulatory peritoneal dialysis (CAPD), but there are significant regional differences. Hong Kong has the highest PD utilization rate globally, while in mainland China, the utilisation of PD has risen steeply (more than tenfold) over the last decade (Yu and Yang, 2015).

Prolonged exposure of the peritoneum to nonbiocompatible peritoneal dialysis fluid (PDF), peritoneal inflammation, uraemic toxins, and other factors can lead to changes in peritoneal morphology and function, resulting in peritoneal dysfunction, ultrafiltration failure, and peritoneal fibrosis (PF), which are major causes of PD withdrawal in patients with ESRD. Therefore, it is particularly important to explore new PF targets and anti-fibrotic strategies. Therapeutic strategies that have been reported in studies to mitigate PF include, fewer bioincompatible solutions, peritoneal resting, use of osmotic metabolizers and addition of cytoprotective agents. The trace element zinc is known to inhibit oxidative stress, which is one of the main mechanisms in the development and progression of PF. To this end, this review summarises and analyses recent research conducted by domestic and foreign scholars on the possible mechanisms through which zinc prevents PF.

## 2 Mechanisms of PF in PD

**FIGURE 1:**
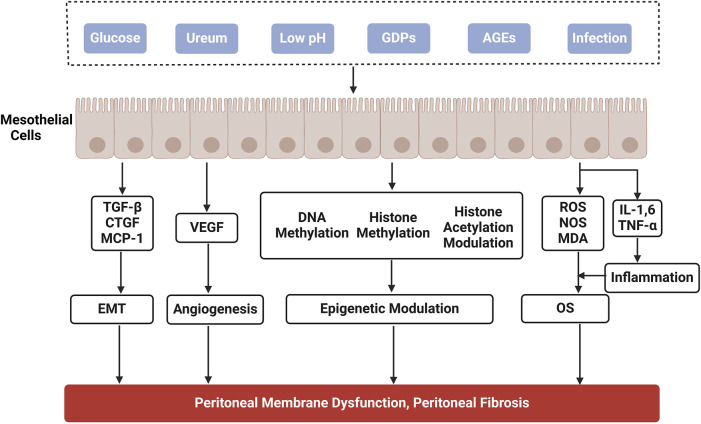
Mechanisms of peritoneal fibrosis in peritoneal dialysis. GDPs, glucose degradation products; AGEs, advanced glycation end products; TGF-β, transforming growth factor-β; CTGF, connective tissue growth factor; MCP-1, macrophage chemoattractant protein-1; VEGF, vascular epithelial growth factor; IL, interleukin; TNF-α, tumor necrosis factor-α; ROS, reactive oxygen species; NOS, nitric oxide synthase; MDA, malondialdehyde; OS, oxidative stress; EMT, epithelial-mesenchymal transition.

PD is considered an effective alternative therapy for patients with ESRD. However, its effectiveness depends on the structural and functional integrity of the peritoneum. PD is mainly accomplished by the bio-semipermeable nature of the peritoneum, which is composed of human peritoneal mesothelial cells (HPMCs). Prolonged exposure of HPMCs to hypertonic PDF, which has a high glucose (HG) concentration, is the main cause of PF. PDF exposes peritoneal tissues to long-term inflammation and oxidative stress (OS), resulting in the accumulation of inflammatory cells, generation of reactive oxygen species (ROS), accumulation of the extracellular matrix (ECM), and neovascularisation, thereby contributing to the formation of PF (Figure 1). Alternatively, damaged PMCs secrete numerous cytokines, such as transforming growth factor-β (TGF-β), connective tissue growth factor (CTGF), and vascular endothelial growth factor (VEGF). Overexpression of these cytokines can further promote peritoneal tissue fibrosis (Liu et al., 2022). With the recognition of the importance of fibrosis in peritoneal dysfunction, it has become increasingly crucial to explore the molecular basis of PF and slow its onset and progression.

### 2.1 Epithelial-mesenchymal transition (EMT)

MCs are of mesodermal origin and share characteristics with both epithelial and endothelial cells, which may undergo EMT and endothelial-to-mesenchymal transition (EndMT), respectively. Recently, several authors have proposed renaming the mesenchymal conversion of MCs with a more appropriate term: mesothelial to mesenchymal transition (MMT), in contexts such as the lung, liver or peritoneum (Kriz et al., 2011; Perez-Lozano et al., 2013). EMT plays an important role in many tissues and organs, promoting tissue repair, but excessive repair can lead to tissue and organ fibrosis. MMT is a key mechanism in PF. The epithelial-mesenchymal transdifferentiation of peritoneal mesothelial cells involves the dissociation of cellular junctions due to the downregulation of intercellular adhesion molecules and loss of apical-basal polarity of microvilli. This enables mesothelial cells to migrate and degrade the basement membrane. Transdifferentiated myofibroblasts secrete abundant extracellular matrix, VEGF, and other cytokines, leading to PF, angiogenesis, and ultimately ultrafiltration failure (Devuyst et al., 2010).

The *in vivo* MMT process involves the integration of diverse signals triggered by multiple factors. It is also accompanied by changes in protein expression, including loss of epithelial marker proteins such as Epithelial Cadherin (E-cadherin), cytokeratin, and increased expression of mesenchymal markers, including vimentin and α- Smooth muscle motor protein (α- SMA).

The adhesion of E-cadherin is crucial for maintaining cell connections. During EMT, E-cadherin expression is inhibited, weakening its adhesion to cells. Consequently, the loss of E-cadherin is a hallmark of EMT (Matos et al., 2016). Studies have identified the transcription factor Snail as a direct and strong repressor of E-cadherin expression (Cano et al., 2000), a process regulated by TGF-β1 (Peinado et al., 2007).

Smad is a downstream protein of TGF-β1. Experiments have shown that when knockout of the Smad3 gene induced a PF model, the peritoneum of mice did not show significant thickening, and PF was reduced (Duan et al., 2014). This demonstrates the key regulatory role of the TGF-β1/Smad3 pathway in PF, which also suggests that inhibiting TGF-β may inhibit the MMT process and suppress PF (Tomino, 2012).

TGF-β1 is a multifunctional cytokine produced mainly by activated macrophages, peritoneal mesothelial cells, and fibroblasts. It is the most important cytokine that directly stimulates peritoneal proliferation. TGF-β1 activates Smad-dependent and Smad-independent pathways. However, most profibrotic actions of TGF-β1 operate via Smad signalling (Lan, 2011), which activates Smad and induces the transcription and expression of downstream target genes in this pathway, directly and indirectly stimulating PF. Additionally, there are non-Smad proteins, such as AKT/mTOR signalling (Patel et al., 2010), C-Jun N-terminal kinase (JNK) (Vavvas et al., 2012), Wnt/β-catenin (Zhang et al., 2013), and integrin ligase kinase/glycogen synthase kinase 3β (ILK/GSK-3β) (Luo et al., 2014) that are involved in EMT. Among them, activation of the Wnt/β-catenin signalling pathway promotes EMT in various epithelial cells, including PMCs. After incubation with LiCl, the phosphorylation of GSK-3β leads HMPCs to undergo EMT characterized by increased expression of a-SMA and decreased expression of E-cadherin, indicating that GSK-3β plays a key role in the EMT of HMPCs (Zhang et al., 2013).

### 2.2 Angiogenesis

Inflammation and injury to the peritoneum contribute to progressive angiogenesis and fibrosis, ultimately leading to ultrafiltration failure. Patients undergoing long-term PD often exhibit vasculopathy and angiogenesis in the peritoneum with the degree of vascularisation related to the area of fibrotic tissue, suggesting the involvement of angiogenesis in PF progression (Zhang et al., 2017). Angiogenesis results from increased production of VEGF and other proangiogenic factors, such as macrophage chemoattractant protein-1 (MCP-1), interleukin (IL)-1β, IL-6, IL-8 and tumour necrosis factor-α (TNF-α), that stimulate the formation of new capillaries in the peritoneum.

Bioincompatible PD solutions, growth factors (epidermal growth factor and TGF-β1), and inflammatory cytokines (IL-1α, IL-6) induce VEGF production (Zhang et al., 2017). Increased VEGF production leads to vasodilatation, increased capillary wall permeability, high peritoneal solute transport, and ultrafiltration failure. Experimental studies have proven that effluent VEGF concentration increases with PD duration, but decreases when patients switch to glucose-free PDF, suggesting that HG levels are associated with increased VEGF production (Mortier et al., 2005). Inhibiting VEGF expression or blocking VEGF-related signalling pathways has also been targeted for PF inhibition.

Ang and its receptor, Tie2, are implicated in tumour development. In a uraemic rat model, peritoneal angiogenesis and fibrosis occurred following PD therapy, accompanied by increased Ang-2 expression and reduced Tie2 expression (Yuan et al., 2009).

### 2.3 Epigenetic modulation

Recent studies have reported the epigenetic modifications associated with PF, and accumulating evidence suggests that epigenetic therapies have the potential to prevent and treat PF. Epigenetics refers to the genetic regulation patterns in which gene expression, which leads to phenotypic variance, is modified without changes in the DNA sequence. Epigenetics mainly includes DNA methylation, histone modification, and noncoding RNA regulation (Bartke and Kouzarides, 2014). DNA methylation is a process by which DNA methyltransferase (DNMT) uses S-methionine as a donor and transfers its methyl group to the 5th carbon atom of the DNA cytosine linked to form 5-methylcytosine (Wang et al., 2020). Currently, five different DNMTs have been identified (DNMT1, DNMT3a, DNMT3b, DNMT31, and DNMT2) (Chen and Zhang, 2020). Kim et al. (2014) found that in rats with experimental encapsulated peritoneal sclerosis, DNMT1 elicited Ras GTPase activating-like protein 1 (RASAL1) hypermethylation and decreased RASAL1 expression, which resulted in upregulation of peritoneal TGF-β1 and increased peritoneal thickness.

Histone methylation, which involves transferring methyl groups to the amino acids of histones and non-histone proteins in the presence of histone methyltransferases, has been implicated in multiple fibrotic diseases, including PF (Aguilera et al., 2017b; Trackman et al., 2018). Several histone methyltransferases, such as G9a, SET7/9, and enhancer of zeste homologue 2 (EZH2), are involved in PF (Aguilera et al., 2017b; Trackman et al., 2018; Shi et al., 2019). Shi et al. (2019) found that EZH2, a key epigenetic regulator of PF, was highly expressed in the peritoneum of mice with PF induced by chlorhexidine gluconate (CG) or HG PDF.

Histone acetylation is a dynamic process regulated by histone acetyltransferases (HATs) and histone deacetylases (HDACs). Several studies have investigated the effects of HDAC inhibitors on PF. For example, inhibition of HDAC6 by Tubastatin A (TA) significantly attenuated PF, suppressed EMT in peritoneal mesothelial cells of mice and reduced the expression of inflammatory cytokines/chemokines (Xu et al., 2017). Aguilera et al. (2017a) found that Anti-fibrotic effects of valproic acid in experimental peritoneal fibrosis (VPA) can significantly ameliorate the upregulation of profibrotic factors (TGF-β, fibronectin, and Smad3), reduce neoangiogenesis and the expression of proinflammatory cytokines (TNF-α, IL-1β, and MCP-1) observed in PF.

In summary, enzymes involved in DNA methylation or histone modification can affect the occurrence and development of PF by regulating DNA methylation, histone methylation, or acetylation, suggesting that pharmacological or genetic interventions targeting the activity or expression of these regulated enzymes may offer a new approach to combat PF.

### 2.4 OS

OS occurs arises primarily from an imbalance between oxidative and antioxidant effects, leading to the production of excessive oxygen radicals, including ROS and reactive nitrogen species. These radicals participate in the pathophysiological processes of various diseases. Patients undergoing PD exhibit excessive OS compared to the general population and patients with predialysis ESRD. Chronic inflammation and OS markers (e.g., glutathione peroxidase and superoxide dismutase activities, total antioxidant capacity [TAC], malondialdehyde [MDA] levels, and protein carbonyl formation) were significantly increased in the blood, urine, and peritoneal fluid of patients undergoing PD compared to healthy controls (Boudouris et al., 2012). Additionally, serum levels of several established OS markers [e.g. thiobarbituric acid-responsive substances (TBARS), MDA, advanced oxidation protein product (AOPP), AGEs, and asymmetric dimethylarginine (ADMA)] were significantly elevated in patients undergoing PD compared to age- and sex-matched healthy controls (Kocak et al., 2008). In addition, patients undergoing PD are significantly deficient in antioxidants, as indicated by lower serum ascorbic acid, vitamin E, and glutathione levels compared to controls (Tarng et al., 2002). These studies show that patients undergoing PD are characterised by increased formation of oxidised molecules and loss of antioxidants compared to the general population and patients with predialysis ESRD. The main source of OS in PD is the non-physiological composition of conventional PD solutions, including an HG concentration, increased osmolarity, and acidic pH (Liakopoulos et al., 2017). During thermal sterilisation of PD fluids, GDPs accumulate in the dialysate. PMCs exposed to HG-containing GDPs induce the generation of AGEs, which bind to the receptor for AGEs (RAGE) and trigger ROS production (Roumeliotis et al., 2020). Accumulating evidence suggests that OS plays a pivotal role in the pathogenesis of chronic PM damage.

#### 2.4.1 Role of antioxidants in PF

Exogenous intake of antioxidants has repeatedly been shown to prevent inflammation and OS in patients with chronic kidney disease (CKD) and those on dialysis (Lupinacci et al., 2018). As the culprits of accelerated OS in PD are the overproduction of ROS and reduced antioxidant activity, some researchers have hypothesised that topical antioxidant supplementation ameliorates OS in these patients (Roumeliotis et al., 2019) and, therefore, may maintain the integrity of the PM or even exert clinical benefits (Roumeliotis Stefanos et al., 2021). Current research on antioxidants in PF is summarised in Table 1.

**TABLE 1:** Summary of studies exploring antioxidants in PF.

Antioxident	Research object	Results	References
NAC	PD rats	prevent the increase of collagen I and hsp 47 in rats omental; inhibit TGF-β1 and VEGF in rats omental	Noh et al. (2006)
angiotensin II receptor blocker/ARB	PD rats	prevent the increase of collagen I and hsp 47 in rats omental; inhibit TGF-β1 and VEGF in rats omental	Noh et al. (2006)
Apolipoprotein A-I (apoA-I)	PD rats	N-cadherin, Fibronectin, Vimentin, and α-SMA expression decreased; increased the activity of SOD and GSH-Px; inhibit expression of IL-6, IL-1β and TNF-α	Lu et al. (2023)
Empagliflozin	PD rats	inhibit the MDA levels, and ROS generation, raise the SOD activity, GSH-Px activity in PD rats; increase the level of Nrf-2 and HO-1 in PD + empagliflozin rats	Shi et al. (2022)
NAC	PD patients	increase serum NAC levels (2.6–24.8 mmol/L); decrease serum IL-6 levels (9.4–7.6 pg/mL)	Nascimento et al. (2010)
Vitamin E	PD patients	decrease the levels of plasma MDA, AOPP, CRP and TNF-α; increase the level of VitE, SOD, GSH-Px and FMD	Zhang (2010)
Vitamin E	PD patients	increased erythrocyte antioxidant enzymes activity and TAC levels, decreased MDA concentration and carbonyl compound formation	Boudouris et al. (2012)
Vitamin C	PD patients	increased erythrocyte antioxidant enzymes activity and TAC levels, decreased MDA concentration and carbonyl compound formation	Boudouris et al. (2012)
25-hydroxyvitamin D	PD patients	decrease hsp,IL-6 and TNF-α, lower MDA concentration, higher SOD and GSH-Px	Zhang et al. (2021)
Zinc	PD patients	decrease plasma MDA, increase the activity of GPx and SOD	Guo et al. (2013)
Zinc	PD patients	increase the levels of IL-1α/β and TNF-α	Kimmel et al. (1996)

NAC, N-Acetylcysteine; TGF-β, transforming growth factor-β; VEGF, vascular epithelial growth factor; α-SMA, α-smooth muscleactin; SOD, superoxide dismutase; GSH-Px, glutathione peroxidase; IL, interleukin; TNF-α, tumor necrosis factor-α; MDA, malondialdehyde; ROS, reactive oxygen species; AOPP, advanced oxidation protein product; CRP, C-reactive protein; FMD, flow mediated dilatation; HO-1, heme oxygenase-1; Nrf2, nuclear factor erythroid-2-related factor 2; TAC, total antioxidant capacity.

## 3 Role and mechanism of zinc in the regulation of PF

### 3.1 Zinc and zinc homeostasis

Zinc is an essential trace element (TEs) in eukaryotes, and it has antioxidant, anti-inflammatory, and antiproliferative properties. Zinc is the second most abundant metal in the body and is unevenly distributed among different organs and tissues. The prostate, pancreas, and bone are considerably high in zinc. In contrast, the heart, brain, and plasma have relatively low concentrations of zinc. Plasma, containing only 1 ug/g, is probably the most important reservoir for zinc homeostasis (Wessels et al., 2017). As a cofactor for over 300 enzymes, zinc plays an important role in several biochemical pathways, including the activation of superoxide dismutase, a powerful enzyme with antioxidant activity (Prasad and Bao, 2019). It is directly involved in nucleic acid and protein synthesis, cell differentiation, proliferation, and many other vital cellular metabolic processes. Zinc deficiency triggers symptoms such as developmental delay, anorexia, skin damage, skeletal deformities, and immune deficiency. Therefore, the homeostatic balance of zinc metabolism is essential for maintaining normal physiological functions in organisms.

Zinc acts as an antioxidant by accelerating the activity of copper/zinc superoxide dismutase, promoting the synthesis of metallothionein (MT) and maintaining membrane structure. Its modulation of cellular oxidative/antioxidant homeostasis involves multiple interrelated events, including (i) regulating oxidant production and metal-induced oxidative damage, (ii) regulating GSH metabolism, and (iii) direct or indirect regulation of redox signalling (Oteiza, 2012). These alterations may lead to changes in organ cellularity, organisation, and connectivity, thereby increasing the risk of disease later in life (Li et al., 2022). In addition, the antioxidants and anticancer mechanisms associated with zinc homeostasis appear to play an inhibitory role in tumour cell growth. Zinc prevents genomic instability and gene mutations.

Previous studies have demonstrated that zinc deficiency triggers OS, oxidant-mediated damage to cellular components, and alterations in cell function and proliferation (Zhang et al., 2013). Zinc supplementation reduces markers of OS and lowers inflammatory cytokines levels and infection incidence (Prasad et al., 2007). Moreover, zinc supplementation in healthy individuals reduces plasma levels of OS-related byproducts, 4-hydroxyalkenals (HAE), MDA, and 8-hydroxydeoxyguanine (Prasad et al., 2004), indicating that zinc supplementation inhibits fibrosis in myocardial, perivascular, liver, and cystic fibrosis (Wang et al., 2005; Takahashi et al., 2007; Gandhi et al., 2008; Van Biervliet et al., 2008; Zhang et al., 2012a).

Zinc is absorbed in the gut and distributed throughout the body via specific transport proteins. Zinc ions cannot pass freely through the cell membrane; thus, specific transporter proteins and membrane channels are involved in zinc transport and metabolism. Zinc transporters (ZnTs) are particularly important among the proteins that maintain zinc homeostasis. ZnT1-7, a member of the cation diffusion facilitator family of metal ion carriers, transports zinc out of the cell or delivers it to organelles and plays an important role in the regulation of intracellular zinc homeostasis (Kambe et al., 2004). ZnTs are not only directly involved in the homeostatic metabolism of zinc ions in cells but also influence the onset and progression of diseases such as tumours or chronic inflammatory diseases through complex mechanisms.

Previous studies confirmed that patients with ESRD have varying degrees of zinc deficiency. Lobo et al. (2013) evaluated plasma zinc levels in 48 patients undergo HD and 20 healthy individuals and confirmed lower zinc levels in the HD population. Studies have also shown that patients with CKD, undergoing HD and PD have lower zinc levels than healthy individuals (Rana, 2014). One of the main pathogenesis of diabetic kidney disease (DKD) is OS caused by ROS in the kidney, and the significance of zinc in preventing and slowing the progression of DKD has been widely evaluated in experimental studies. A previous study indicated that zinc pre-treatment provides effective protection against HG-induced EMT in renal tubular epithelial cells by the reduced upregulation of α-SMA and vimentin and ameliorated expression of E-cadherin (Zhang et al., 2015). In a study of PF in PD, ZnT5 and ZnT7 induced by HG were found to protect rat peritoneal mesothelial cells from apoptosis (Zhang et al., 2013).

### 3.2 Role of the TE zinc in PF

It is currently believed that OS and chronic inflammation may play important causative roles in many chronic diseases, including PF in PD. Zinc deficiency increases levels of inflammatory cytokines and OS, induces apoptosis, and leads to cellular dysfunction.

Zinc pre-treatment significantly attenuated HG-induced ROS production in rat peritoneal mesothelial cells (RPMCs) by activating the PI3K/Akt and mitogen-activated protein kinase (MAPK)/ERK signalling pathways to inhibit apoptosis in RPMCs (Zhang et al., 2012a). Another study also noted a significant positive correlation between the serum copper/zinc ratio and OS in patients with uraemia (Guo et al., 2009). Guo et al. (2011) found that patients with CAPD show significantly higher copper/zinc ratios compared to healthy individuals, with this ratio being strongly associated with inflammation, OS, and immune dysfunction. Fan et al. (2017) showed that zinc inhibits HG-induced ROS production in peritoneal mesothelial cells. Altogether, the abundant evidence suggests that TEs have the potential to ameliorate PF by improving the inflammatory response and OS status in patients with CAPD.

Zinc may be involved in host defence by maintaining the structure and function of cell membrane barriers. Several studies have examined the effects of zinc depletion and supplementation on endothelial cell barrier permeability. The intestinal epithelial barrier consists of intercellular junction complexes between neighbouring cells, including tight and adhesive junctions. Zinc deficiency disrupts the epithelial cell barrier by disrupting these junctions through multiple mechanisms. One way in which zinc affects structural proteins is through enhanced degradation of E-cadherin and β-catenin (Finamore et al., 2008). This is also observed in zinc-deficient airway epithelial cells, where protein hydrolysis of E-cadherin and β-catenin is accelerated, leading to structural and functional disruptions (Bao and Knoell, 2006). Hypozincemia induces neutrophil migration by increasing chemokine production. Aggravated inflammation may develop and lead to mucosal damage, potentially leading to intestinal and pulmonary diseases. Conversely, zinc supplementation protects and restores membrane function and structure (Finamore et al., 2008). Furthermore, previous studies indicate that zinc can attenuate HG-induced upregulation of -SMA and collagen I and ameliorate E-cadherin expression in the RPMCs, suggesting that zinc may inhibit fibrosis via reversing EMT in the RPMCs (Zhang et al., 2012b).

Zinc supplementation protects various cell types from OS by maintaining cell membrane stability and activating the PI3K/AKT pathway. Due to its redox-regulated properties, zinc plays an indispensable role in enzymatic reactions and influences redox-regulated signalling pathways, like the nuclear factor erythroid-2-related factor 2 (Nrf2) pathway. Consequently, the crosstalk between micronutrients and redox signalling pathways is currently a hot area of research.

### 3.3 Zinc as a modulator of the keap1/Nrf2/ARE signaling pathway

#### 3.3.1 Keap1/Nrf2/ARE system

The transcription factor Nrf2 is a central regulator of redox reactions, metabolism, and protein homeostasis, intersecting with many other signaling cascades (Dodson et al., 2019). Under normal physiological conditions, Nrf2 activity is tightly regulated by Kelch-like ECH-associated protein 1 (Keap1). Nrf2 forms a complex with Keap1 in the cytoplasm, which is then degraded by the ubiquitin-proteasome pathway. On exposure to excessive ROS, Keap1 is inactivated, allowing Nrf2 to liberate and translocate into the nucleus. There, Nrf2 binds to the antioxidant response element (ARE) and initiates the expression and transcription of downstream antioxidant genes, including haeme oxygenase-1 (HO-1), NAD(P)H dehydrogenase quinone 1 (NQO1), and the modulatory and catalytic subunits of γ-glutamyl cysteine ligase (GCLM and GCLC, respectively) (Jaiswal and Jaiswal, 1998). Nrf2 protects against OS and inflammation. Numerous studies have shown that Nrf2 plays a pivotal role in inflammatory diseases that affect different systems, including gastritis, colitis, pneumonia, arthritis, cardiovascular disease, liver ischemia-reperfusion injury (Yi et al., 2020), neurodegenerative disease, and brain damage (Ahmed et al., 2017).

Nrf2 mediates the transcriptional activation of antioxidant enzyme genes such as HO-1, NQO1, and those belonging to the glutathione s-transferase family. According to a previous study, the activation of Nrf2 and its downstream gene HO-1 plays an antioxidant role in several cell lines (Nguyen et al., 2009). Nrf2-mediated HO-1 upregulation reduced inflammation by increasing the efferocytic activity of macrophages in a mice model treated with TauCl (taurine chloramine) (Kim et al., 2015). In renal diseases, Aristolactam I has been shown to induce ferroptosis of HK-2 by inhibiting the Nrf2-HO-1/GPX4 signalling pathway (Deng et al., 2021). Therefore, the Keap1/Nrf2/ARE system, one of the most important antioxidant pathways in eukaryotic cells, is a defence mechanism against OS and plays a key role in the pathogenesis and progression of many diseases.

#### 3.3.2 Effect of zinc on Nrf2/ARE pathway

Nrf2-regulated genes are continuously increasing and include genes involved in antioxidant defence, glutathione synthesis, NADPH regeneration, drug interaction, and metabolic regulation, including lipid and carbohydrate metabolism (Hayes and Dinkova-Kostova, 2014). Nrf2 has previously been shown to be regulated by changes in the cellular state of individual TEs, such as zinc.

Exogenous zinc intervention may improve the total cellular antioxidant capacity and attenuate OS induced by GDPs, HG levels, and inflammation by promoting the nuclear translocation of Nrf2 and upregulating the expression of the downstream target genes HO-1 and NQO1, which are important components of human antioxidant enzymes. Keap1 acts as a sensor of intracellular zinc. Zinc binding triggers a conformational switch in the cullin3 substrate adaptor function of Keap1, which stabilises Nrf2 and can activates the transcription of target genes (McMahon et al., 2018). Furthermore, zinc modulates the activity of several kinases and phosphatases, thereby enhancing Nrf2 activity (Schwarz et al., 2019).

**FIGURE 2:**
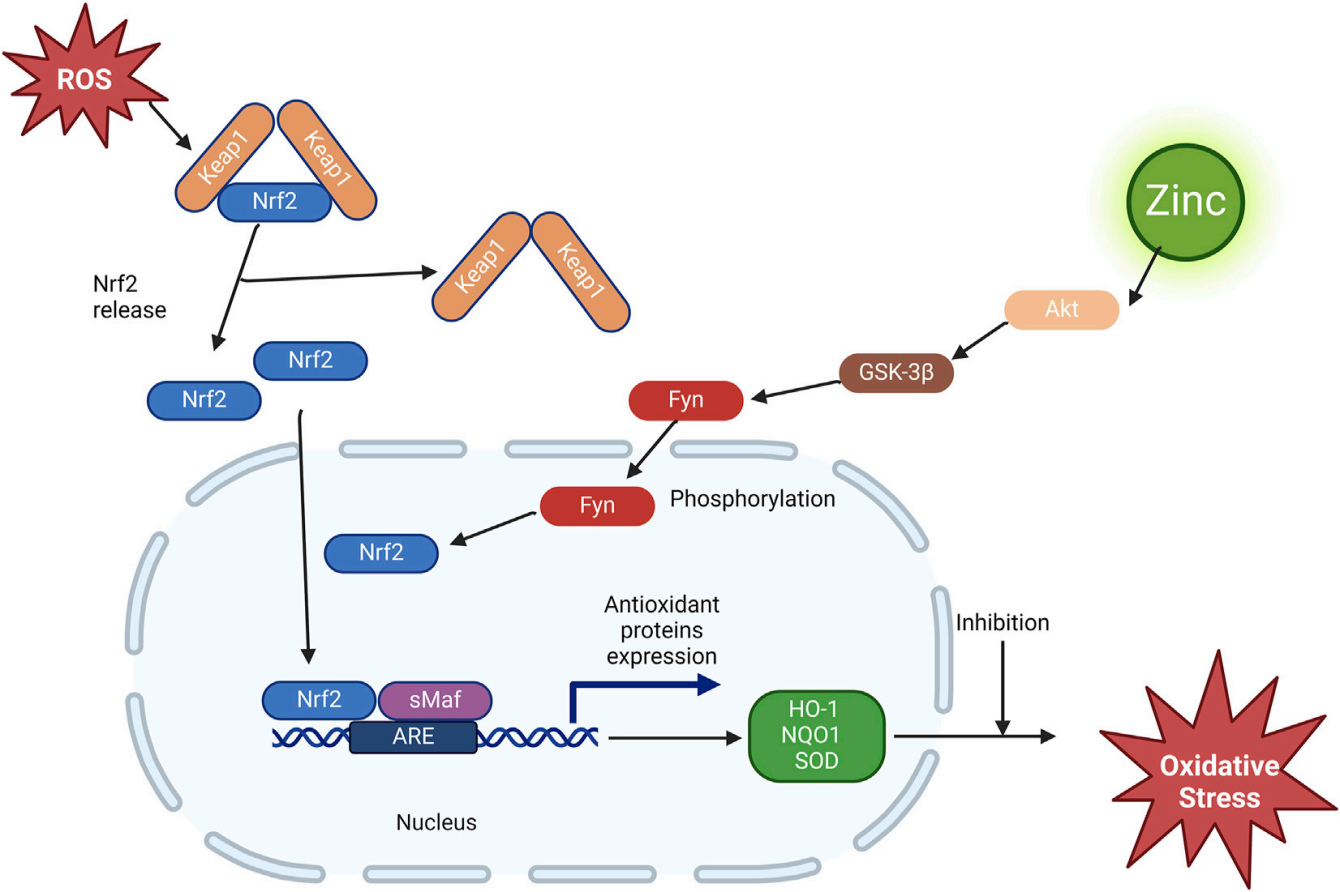
Effect of zinc on the Nrf2/ARE pathway. ROS, reactive oxygen species; Nrf2, nuclear factor erythroid-2-related factor 2; Keap1, Kelch-like ECH-associated protein 1; sMaf, smooth muscle activating factor; ARE, antioxidant response element; GSK-3β, glycogen synthase kinase three beta; HO-1, heme oxygenase-1; NQO1, NAD(P)H dehydrogenase quinone 1; SOD, superoxide dismutase.

Zinc induces Nrf2 upregulation through Akt-mediated prevention of Fyn nuclear translocation, exerting antioxidant effects (Figure 2). Previous studies have shown that zinc negatively regulates Akt-negative regulators protein tyrosine phosphatase (Haase and Maret, 2005a; b) and PTEN (Wu et al., 2003). Therefore, it has been suggested that zinc-stimulated Akt phosphorylation and, consequently, glycogen synthase kinase three beta (GSK-3β) phosphorylation may reduce Fyn nuclear translocation, leading to the export of Nrf2 to the cytosol. This was confirmed in a study of renal tubular epithelial cells under *in vitro* diabetes-mimicking conditions (Li et al., 2014). Fyn is a well-known negative regulator of Nrf2 that enters the cytoplasm to export Nrf2 for degradation (Kaspar and Jaiswal, 2010). The activated GSK-3β phosphorylated Fyn at threonine residues, leading to nuclear localisation of Fyn and Fyn phosphorylated tyrosine 568 of Nrf2. Fyn phosphorylation led to the nuclear accumulation of Fyn, and Nrf2 phosphorylation resulted in Nrf2’s nuclear export, ubiquitination, and degradation (Jain and Jaiswal, 2006).

#### 3.3.3 Zinc as a modulator of the Keap1/Nrf2/ARE signaling pathway in PF

Currently, available research data indicate that TE zinc effectively regulates the antioxidant signalling pathway-Nrf2 in the pathogenesis of various diseases. This was confirmed in subsequent studies.

Zinc-mediated Nrf2 hyperexpression has been studied in various diseases (Kim and Vaziri, 2010; Mehta et al., 2011), particularly in mouse fibroblastic cells, as shown by McMahon et al. (2010), supporting the protective role of zinc against OS. Yang et al. (2017) showed a protective role of zinc in diabetic rats through an increase in Nrf2, GSK-3β phosphorylation, and protein Kinase B. A study at our centre investigated how zinc increases Nrf2 transcription and attenuates DKD *in vivo*, in diabetic experimental models and *in vitro* in human renal tubule cells (Li et al., 2014). Zinc deficiency enhanced diabetes-induced liver injury in type 1 diabetic mice, likely due to downregulation of Akt-GSK3β-Nrf2-mediated antioxidative function (Xu et al., 2012). In an *in vitro* spinal cord injury (SCI) model, Li et al. (2020) found that zinc treatment promoted motor function and neuronal recovery after SCI. Simultaneously, zinc reduces the levels of ROS and malondialdehyde in the spinal cord tissue after SCI and increases the levels of Nrf2, HO-1, and HQO1 in the nucleus, indicating that zinc can reduce OS through the Nrf2/HO-1 pathway in spinal cord tissue. Kaufman et al. (2020) observed that zinc deficiency impairs the capacity of human IMR-32 neuroblastoma cells to upregulate HO-1 and activate the Nrf2 pathway, leading to a higher sensitivity of IMR-32 cells to DA cytotoxicity. Another study found that zinc supplementation activates Nrf2 signalling in human coronary smooth muscle cells and attenuates hypoxia/reoxygenation-induced ROS production, which provides a basis for exploiting therapeutic drugs for the treatment of coronary heart disease (Yang et al., 2023). In a rat model of osteoarthritis, zinc supplementation blocked OS, lowered GSH levels, decreased HO-1, IL-10, IL-1β, and matrix metalloproteinase (MMP)-13 expression, and increased Nrf2 and phosphorylated-Akt expression (Huang et al., 2018). This suggests that zinc protects articular chondrocytes through changes in Nrf2-mediated antioxidants, cytokines, and MMPs.

OS plays a pivotal role in the pathogenesis of peritoneal dialysis-associated PF, including inflammation, HG, and other non-biocompatible components; thus, alleviating OS in peritoneal mesothelial cells is crucial for preventing and treating PF. Upon exposure to excessive ROS, Nrf2 can dissociate from Keap1 and accumulate in the nucleus, subsequently binding to antioxidant-responsive element sequences, activating antioxidant-related genes, including NAD(P)H dehydrogenase, NQO1, and HO-1 (McMahon et al., 2010; Wang et al., 2013).

Zinc supplementation attenuates HG-induced ROS generation in peritoneal mesothelial cells (Zhang et al., 2012b). These results indicate that HG induces the expression of nuclear Nrf2 and Nrf2 pathway target genes, indicating activation of the Nrf2 pathway through ROS production (Gao et al., 2019). Notably, zinc supplementation further promotes the expression of nuclear Nrf2 and target genes of the Nrf2 pathway, which is consistent with the findings of Kim and Vaziri (2010), thereby synergistically reducing OS in human peritoneal mesothelial cells. Zinc inhibits HG-induced NLRP3 inflammasome activation and ROS generation by activating the Nrf2 antioxidant pathway in HPMCs to attenuate PF (Fan et al., 2017). These results suggest that zinc supplementation may delay PF in PD by activating the Nrf2 pathway and subsequently decreasing OS, offering new perspectives for the research in PF prevention and treatment.

### 3.4 Crosstalk between zinc and other signaling pathways in PF

**FIGURE 3:**
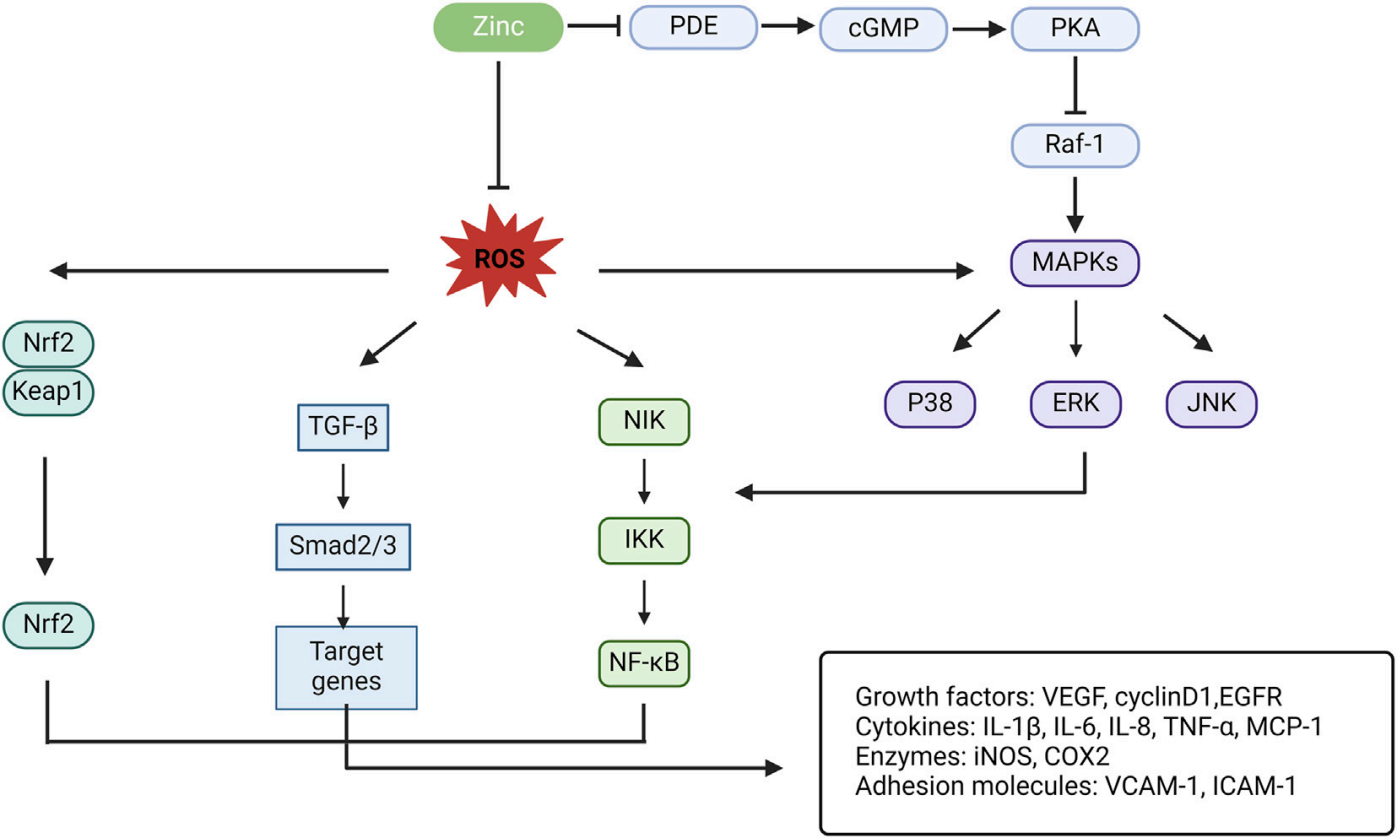
Schematic illustration for the crosstalk of zinc and other signal pathways in PF. PDE, phosphodiesterase; cGMP, cyclic guanosine monophosphate; PKA, protein kinase A; Raf-1, a serine/threonine kinase; MAPK, mitogen-activated protein kinase; ERK, extracellular signal-regulated kinases (¼ mitogen-activated protein kinases); JNK, c-Jun N-terminal kinase; NIK, nuclear factor-kB inducible kinase; IKK, Ik kinase; NF-kB, nuclear factor kappa B; TGF-β, transforming growth factor-β; Nrf2, nuclear factor erythroid-2-related factor 2; Keap1, Kelch-like ECH-associated protein 1; VEGF, vascular epithelial growth factor; EGFR, epithelial growth factor receptor; IL, interleukin; TNF-α, tumor necrosis factor-α; iNOS, inducible nitric oxide synthase; MCP-1, macrophage chemoattractant protein-1; COX2, cyclo-oxygenase 2; VCAM-1, vascular cell adhesion molecule-1; ICAM-1, intracellular adhesion molecule-1.

Signalling pathways coordinate communication between the cell surface and nucleus, among different cells, and between cells and the ECM. Crosstalk between the TE zinc and signalling pathways is also pivotal in the mechanism of PF (Figure 3). This cellular crosstalk is very complex and remains largely unknown. Efforts should be made to elucidate the intricacies of these complex processes to help develop better preventive and therapeutic strategies.

#### 3.4.1 Nuclear factor kappa B (NF-κB)

NF-κB is a widespread nuclear transcription regulatory protein that regulates the expression of multiple genes involved in inflammation, immunity, cell proliferation, differentiation, and apoptosis. Chronic activation and aberrant regulation of the NF-κB signalling pathway are major mechanisms in the development of many diseases.

EMT and MMT are important pathogenic mechanisms in peritoneal dialysis-associated PF. The NF-κB signalling pathway is critical for the induction and maintenance of EMT, including the activation of EMT-specific genes (Huber et al., 2005). It has been shown that parthenolide, an NF-κB inhibitor, primarily exerts its biological activity in inflammatory and tumour diseases through inhibition of NF-κB and targeting multiple steps in the NF-κB signalling pathway. Inhibiting NF-κB may limit EMT-related events in PF (Strippoli et al., 2008; Zhang et al., 2020).

Recent research indicates that zinc modulates NF-κB signalling at various levels. Zinc is involved in inhibiting NF-κB activation in various cells, although the precise mechanism remains controversial. A growing body of literature has confirmed the major role of zinc as a negative regulator of the NF-κB pathway. The zinc finger protein (A20), a key negative regulator of NF-κB activity, acts as a cellular protector against TNF-α-induced NF-κB toxicity. Zinc decreases the levels of cytokines, ROS, and polysaccharides by increasing the concentrations of A20 and peroxisome proliferator-α to regulate NF-κB transcription (Prasad, 2014; Olechnowicz et al., 2017).

Additionally, zinc chelation has been found to increase the DNA binding of NF-κB and AP-1, while zinc supplementation decreases their DNA binding activities. Conversely, another study showed that zinc supplementation increases the DNA-binding activity of NF-κB and AP-1 (Kim et al., 2003). Zhang et al. showed that zinc supplementation reduces HG-induced total NF-κB levels. Furthermore, NF-κB activation was increased in zinc-deficient-stimulated RPMC compared to zinc-sufficient-stimulated RPMC (Zhang et al., 2012b).

#### 3.4.2 TGF-β

TGF-β is a multifunctional cytokine that exerts biological effects by regulating cell function through cell surface-specific receptors and complex signalling pathways. It is the most potent fibrogenic factor identified to date. *In vitro* studies have demonstrated that PMCs can secrete TGF-β. HG PDF stimulates PMCs to produce TGF-β in a dose- and time-dependent manner. In addition to its direct effects, TGF-β can influence other cytokines through signalling pathways such as protein kinase C (Ha et al., 2001) and the renin-angiotensin system to amplify its effects. The role of TGF-β in PF is summarised as follows: stimulating ECM synthesis by PMCs, regulating ECM-degrading enzymes and their inhibitors, and inhibiting mesothelial cell proliferation (Park et al., 2007), which ultimately leads to the formation of PF and failure of peritoneal ultrafiltration.

A previous study showed that zinc deficiency leads to TGF-β1-induced neurogenesis, which controls neuronal precursor cell proliferation and survival by regulating p53-dependent molecular mechanisms (Corniola et al., 2008). Another study indicated that TGF-β1 has both stimulatory and inhibitory effects on osteoclast-like cell formation in mouse marrow cultures, with zinc inhibiting its stimulatory effect (Yamaguchi and Kishi, 1995). Zinc supplementation reduces ethanol- and acetaldehyde-induced liver stellate cell activation, partly by inhibiting Smad signalling (Szuster-Ciesielska et al., 2009). Under HG conditions, zinc supplementation significantly reduces TGF-β1 production and inhibits Smad3 phosphorylation in RPMCs, suggesting that zinc blocks EMT via the Smad pathway.

#### 3.4.3 Mitogen-activated protein kinase (MAPK)

The MAPK signalling pathways currently identified in mammalian cells are a family of serine/threonine kinases containing mainly extracellular signal-regulated protein kinases, c-Jun N-terminal kinases, and P38 MAPK. Activation of P38 MAPK has been associated with increased cellular hypertrophy, apoptosis, proliferation, and inflammation in the heart and liver (Li et al., 2005; Wang et al., 2011). Zinc deficiency also activates P38 MAPK-mediated inflammation (Zago MP et al., 2005). In a study on obesity-related renal diseases, Luo et al. demonstrated that zinc inhibits the activation of P38 MAPK and its downstream inflammatory cytokines in renal tubular cells. These results suggest that zinc can delay the progression of obesity-related kidney disease by downregulating P38 MAPK-mediated inflammation (Luo et al., 2016). Recent studies have confirmed that the MAPK signalling pathway plays an important role in the transdifferentiation of peritoneal mesothelial cells. One study investigated the effects of zinc on MAPK (JNK and P38) expression in HG-treated RPMCs. The results showed that zinc significantly reduced phosphorylated JNK and P38 expression, while zinc deficiency had the opposite effect on HG-treated RPMCs, suggesting that zinc may inhibit EMT by suppressing the MAPK pathway (Zhang et al., 2012b).

## 4 Summary

PF is a significant complication in patients undergoing PD and one of the most important reasons for withdrawal from PD in patients with ESRD. Prevention and treatment of PD-associated PF are crucial for patients undergoing PD. As an antioxidant, TE zinc can exert its anti-OS capacity to delay PF progression. Developing zinc-containing PDF or administering oral zinc preparations to high-risk PD patients to verify whether zinc can alleviate PF will be the focus of our future research. Additionally, zinc can influence PF through multiple signalling pathways, including Nrf2, NK-κB, TGF-β, and MAPK. A full understanding of the role of zinc in PF could enhance the protection and functional integrity of the peritoneum during long-term PD. Future studies and explorations in animals and humans are needed to provide a new perspective on the prevention of PF to maintain peritoneal membrane function for an extended period in patients undergoing PD.
